# Supplementary material for: Chronic Kidney Disease-Induced Arterial Media Calcification in Rats Prevented by Tissue Non-Specific Alkaline Phosphatase Substrate Supplementation Rather Than Inhibition of the Enzyme
Source: Pharmaceutics. 2021 Jul 26;13(8):1138. doi: 10.3390/pharmaceutics13081138 (PMC8399849; doi:10.3390/pharmaceutics13081138)
Supplement: Supplementary file 1 [file pharmaceutics-13-01138-s001.zip › pharmaceutics-1276941-supplementary.pdf]

pharmaceutics

MDPI

Supplementary Materials: Chronic kidney disease induced-arterial media calcification in rats can be prevented by tissue non-specific alkaline phosphatase substrate supplementation rather than enzyme inhibition

Britt Opdebeeck, Ellen Neven, José Luis Millán, Anthony B Pinkerton, Patrick C D'Haese and Anja Verhulst

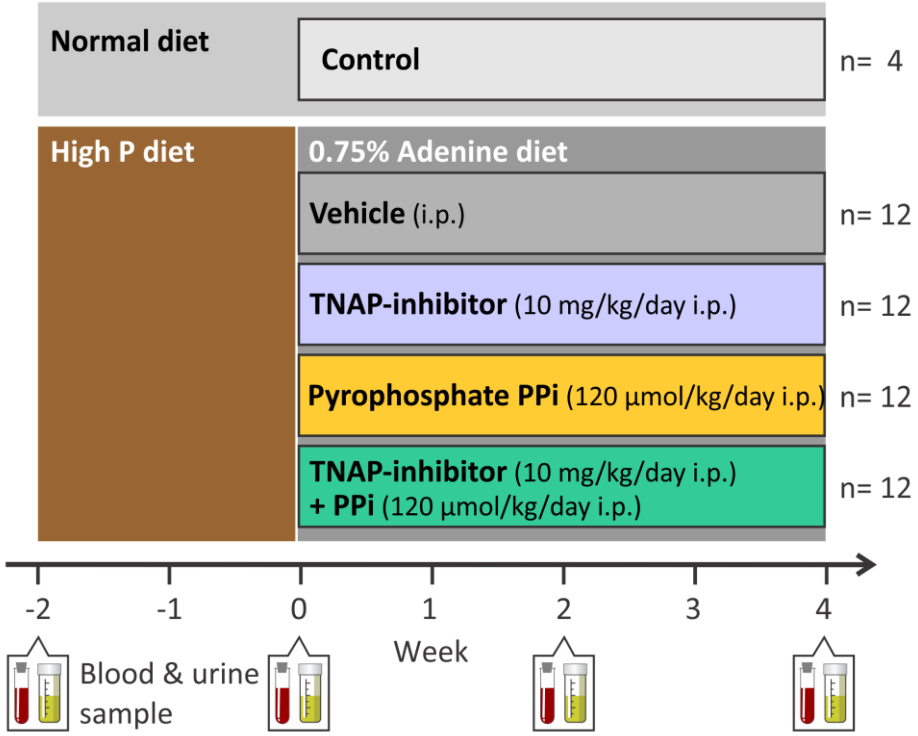

Figure S1. Study setup.

Table S1. Target sequences of TaqMan primers.

Gene	Target sequence	Product code
Gapdh	accatcttc caggagcgag atcccgctaa catcaaatgg ggtgatgctg gtgctgagta tgtcgtggag tctactggcg tcttcacc	Rn99999916_s1
Tnap	caggatt gaccacgggc accatgaagg caaggccaag caggcgctgc atgaggccgt ggagatggat g	Rn01516028_m1
Sm22-alpha	gaagaaagcc caggagcata agagggagtt cacagacagt caactgcagg aggggaagca cgtca	Rn01642285_g1
Runx2	ggacgaggc aagagtttca ctttgaccat aacggtcttc acaaatcctc cccaagtggc cacttaccac agagctatta aagtgac	Rn01512298_m1
Ntpd1	ggcat tttctgcttt ctactttgtg atggactttt ttaagaagat ggcgaacgac agtgtctcct ctcaggagaa aatgactgag ataacaaaaa acttttgctc	Rn00574887_m1
Npp1	gg aaacaaacca gcacttccgg ccttatctga aacacttctt acccaagcgc ttacactttg ctaaaaatga caggattgag ccact	Rn01638706_m1
Npp3	cgg gagacacatc atctcttcct cccacagttc cagactgtct gcgggctgat gtcagggttg atcc	Rn00571329_m1
Phospho1	agggtc ttggatgtgc cagcgaccct ggctgtggcc cgctaatcag cctctccctg gc	Rn01496968_m1
Sox 9	atct cagggtccac cgaccccacc caccactccc aaaacagacg tgcaagctgg gaaagttgat ctgaaacgag aggggcgccc tctggcagag ggtggcag	Rn01751070_m1
Pit-1	aggcagat ccagttccta atggcttacg agctttaccc attttttatg cctgcacaat tggaatcaac ctcttttcca ttatgtatac tggagcacca ttgctgggc	Rn00579811_m1
Pit-2	ggctcagc ggtctggcag ttgatcgcgt ccttcctgag actcccaatc tcagggacac actgcatcgt gggctctacc attggcttct cgctcgtg	Rn00568130_m1

Table S2. Explanation and calculation of static and dynamic bone parameters.

Parameter	Description	Formula
Bone area	% of tissue area including mineralized bone and osteoid	$\frac{\text{bone area}}{\text{total area}}$
Osteoid area	% of bone area including osteoid	$\frac{\text{osteoid area}}{\text{bone area}}$
Osteoid width	Average width of the osteoid seams	$\frac{\text{osteoid area}}{\text{osteoblast perimeter}} \times 1000$
Eroded perimeter	% of total bone perimeter, eroded by osteoclasts	$\frac{\text{eroded perimeter}}{\text{osteoid perimeter} + \text{eroded perimeter+quiesc}}$
Osteoblast perimeter (relative to total perimeter)	% of total perimeter covered by active osteoblasts	$\frac{\text{osteoblast perimeter}}{\text{osteoid perimeter} + \text{eroded perimeter+quiesc}}$
Osteoclast perimeter (relative to total perimeter)	% of total perimeter covered by resorbing osteoclasts	$\frac{\text{osteoclast perimeter}}{\text{osteoid perimeter} + \text{eroded perimeter+quiesc}}$
Mineral apposition rate (MAR)	Rate by which osteoid is mineralized, calculated as the average distance between two tetracycline labels divided by the time interval between the administration of these labels	$\frac{\text{inter-label distance}}{\text{inter-label time}}$
Adjusted apposition rate	Mineral apposition rate averaged over the entire osteoid surface	$\text{MAR x} \frac{(\text{double labbeled perimeter} + \frac{1}{2}\text{single labbeled perimeter})/1000}{\text{osteoid perimeter} \times \text{total perimeter}}$
Bone formation rate	Area of bone formed per unit of time and existing bone	$\text{MAR} \times \frac{\left(\text{double labbeled perimeter} + \frac{1}{2}\text{single labbeled perimeter}\right)}{\text{total area}}$
Mineralization lag time	Time interval between deposition of osteoid and its subsequent mineralization, averaged over the entire life span of the osteoid seam	$\frac{\text{osteoid width}}{\text{adjusted apposition rate}}$

Table S3. Biochemical aspects of TNAP in plasma of human, mouse and rat.

		**Human**		**Mouse**		**Rat**	
		pH 7.5	pH 9.0	pH 7.5	pH 9.0	pH 7.5	pH 9.0
$K_{i,Pi}$ (mM)		0.8	16.7	1	20	0.24	2.2
Normal	[P_i], mM	0.8–1.44		2.54		2	
	%TNAP inhibition	50–64%	4.5–8%	72%	11%	89%	48%
CKD	[P_i], mM	1		2.66		6	
	%TNAP inhibition	56%	6%	73%	12%	96%	73%

	Activity (OD/hour)	Human plasma	Mouse serum	Rat plasma
pH 7.5	Total phosphatase	0.069 ± 0.001	0.411 ± 0.013	0.166 ± 0.035
	TNAP (inhibited by SBI-425)	0.063 ± 0.003	0.321 ± 0.024	0.000 ± 0.048
	Non-TNAP (residual)	0.006 ± 0.002	0.090 ± 0.011	0.166 ± 0.013
pH 9.8	Total phosphatase	1.459 ± 0.213	6.91 ± 0.091	0.426 ± 0.04
	TNAP (inhibited by SBI-425)	1.204 ± 0.315	6.753 ± 0.115	0.199 ± 0.066
	Non-TNAP (residual)	0.255 ± 0.103	0.157 ± 0.024	0.227 ± 0.026

(Upper table) The Ki values for inorganic phosphate (Pi) were obtained from [1]. Based on the Ki values, percentages of plasma TNAP inhibition were calculated. This historical data suggests that, specifically during CKD with Pi values of 6mM, in rats 96% of plasma TNAP activity is inhibited while in humans and mice with CKD the percentage of TNAP inhibition was lower. Furthermore, it is known that in general systemic TNAP activity is very low since calcium is an inhibitor of TNAP in the micromolar range [2] and under physiological conditions (pH 7.5), TNAP is inhibited more than 90% of its total activity (measured in ideal conditions at pH 9). (Lower table) Total phosphatase refers to the total alkaline phosphatase activity including all isoforms germ-cell, intestinal and tissue non-specific alkaline phosphatase. The total alkaline phosphatase activity was determined in absence (total phosphatase value) and in presence of saturating concentrations of SBI-425 (non-TNAP value). The difference between both values is assigned to the amount of TNAP activity. The total alkaline phosphatase activity was measured via colorimetric analysis using *p*-nitrophenyl phosphate as a substrate which is converted by alkaline phosphatase to a yellow chromogen *p*-nitrophenyl.

Table S4. Kruskal-Wallis test multiple comparisons, *p*-values of biochemical parameters.

	Bonferroni corrected *p*Value								
	**Serum phosphorus**	**Serum creatinine**	**Creatinine Clearance**	**Serum calcium**	**Serum FGF23**	**Serum ALP**	**Aortic ALP**	**Serum AST**	**Serum ALT**
Control vs. Vehicle	0.0203	0.0094	0.0613	0.0117	0.0630	0.1103	>0.9999	0.6128	0.1155
Control vs. TNAPi	0.0081	0.0226	0.0026	0.0956	0.0005	>0.9999	>0.9999	0.7964	0.0327
Control vs. PPi	0.0220	0.0201	0.0306	0.0025	0.2005	0.0814	0.7163	>0.9999	0.1602
Control vs. TNAPi + PPi	0.0060	0.0047	0.0037	0.0269	0.0014	>0.9999	0.0754	>0.9999	0.1141

Table S5. Kruskal-Wallis test multiple comparisons, P-values of arterial calcification.

	Bonferroni corrected *p Value*			
	**Calcium Aorta**	**Calcium a. carotis**	Calcium a. femoralis	Von Kossa aorta
Control vs. Vehicle	0.0319	0.0392	0.1034	0.0764
Control vs. TNAPi	0.0002	0.0016	0.0018	0.0003
Control vs. PPi	0.3813	0.5142	0.3678	>0.9999
Control vs. TNAPi + PPi	0.1222	0.3337	0.3903	>0.9999
TNAPi vs. Vehicle	0.1315	0.6484	0.2850	0.0925
TNAPi vs. PPi	0.0017	0.0180	0.0326	<0.0001
TNAPi vs. TNAPi + PPi	0.0157	0.0363	0.0395	0.0003

Table S6. Kruskal-Wallis test multiple comparisons, *p*-values of aortic mRNA expression.

	Bonferroni corrected *p* Value								
	Tnap	***Npp1***	***Npp3***	***Phospho1***	***Sm22-alpha***	***Sox 9***	***Pit-1***	***Pit-2***	***Runx2***
Control vs. Vehicle	0.0454	0.7742	0.2918	>0.9999	0.0052	0.3638	0.0394	0.0298	>0.9999
Control vs. TNAPi	0.3494	0.1486	0.0289	0.9260	0.0033	0.0455	0.0208	0.0099	0.0925
Control vs. PPi	0.1300	0.9998	0.4751	>0.9999	0.0308	0.8313	>0.9999	0.0371	>0.9999
Control vs. TNAPi + PPi	>0.9999	>0.9999	0.3098	>0.9999	0.0476	0.2916	0.7666	0.0390	>0.9999
Vehicle vs. TNAPi	>0.9999	0.5241	0.7730	0.6601	>0.9999	0.8562	>0.9999	>0.9999	0.0287
Vehicle vs. PPi	>0.9999	0.6617	>0.9999	0.4858	>0.9999	>0.9999	0.0056	>0.9999	>0.9999
Vehicle vs. TNAPi + PPi	0.8131	0.6442	>0.9999	0.6950	0.8411	>0.9999	0.1898	>0.9999	>0.9999
TNAPi vs. PPi	>0.9999	0.0324	0.3890	0.0195	0.9763	0.2138	0.0015	>0.9999	0.0342
TNAPi vs. TNAPi + PPi	>0.9999	0.0301	0.6310	0.0362	0.6761	0.9179	0.0912	>0.9999	0.1749

References:

1. Herries, D.G. Alkaline phosphatase: by R B McComb, G N Bowers, Jr and S Posen. pp 986. Plenum Press, New York and London. 1979. $75 ISBN 0-306-40214-9. *Biochem. Educ.* **1981**, *9*, 76, doi:https://doi.org/10.1016/0307-4412(81)90191-6.
2. Villa-Bellosta, R. Impact of magnesium:calcium ratio on calcification of the aortic wall. *PloS one* **2017**, *12*, e0178872-e0178872, doi:10.1371/journal.pone.0178872.
